# Top and Side Lighting Induce Morphophysiological Improvements in Korean Ginseng Sprouts (*Panax ginseng* C.A. Meyer) Grown from One-Year-Old Roots

**DOI:** 10.3390/plants12152849

**Published:** 2023-08-02

**Authors:** Jingli Yang, Jinnan Song, Jayabalan Shilpha, Byoung Ryong Jeong

**Affiliations:** 1 Shandong Facility Horticulture Bioengineering Research Center, Jia Sixie College of Agriculture, Weifang University of Science and Technology, Shouguang 262700, China; yangjl940123@wfust.edu.cn (J.Y.); jinnansong93@gmail.com (J.S.); 2 Department of Horticulture, Division of Applied Life Science (BK21 Four), Graduate School of Gyeongsang National University, Jinju 52828, Republic of Korea; 3 Institute of Agriculture and Life Science, Gyeongsang National University, Jinju 52828, Republic of Korea; shilphajayabalan@gmail.com; 4 Research Institute of Life Science, Gyeongsang National University, Jinju 52828, Republic of Korea

**Keywords:** ginseng cultivation, lighting direction, morphology improvement, one-year-old root, *Panax ginseng* C.A. Meyer, photosynthesis, phototropism, plant growth chamber

## Abstract

Nowadays, not only the roots, but also leaves and flowers of ginseng are increasingly popular ingredients in supplements for healthcare products and traditional medicine. The cultivation of the shade-loving crop, ginseng, is very demanding in terms of the light environment. Along with the intensity and duration, light direction is another important factor in regulating plant morphophysiology. In the current study, three lighting directions—top (T), side (S), or top + side (TS)—with an intensity of 30 ± 5 μmol·m^−2^·s^−1^ photosynthetic photon flux density (PPFD) were employed. Generally, compared with the single T lighting, the composite lighting direction, TS, was more effective in shaping the ginseng with improved characteristics, including shortened, thick shoots; enlarged, thick leaves; more leaf trichomes; earlier flower bud formation; and enhanced photosynthesis. The single S light resulted in the worst growth parameters and strongly inhibited the flower bud formation, leading to the latest flower bud observation. Additionally, the S lighting acted as a positive factor in increasing the leaf thickness and number of trichomes on the leaf adaxial surface. However, the participation of the T lighting weakened these traits. Overall, the TS lighting was the optimal direction for improving the growth and development traits in ginseng. This preliminary research may provide new ideas and orientations in ginseng cultivation lodging resistance and improving the supply of ginseng roots, leaves, and flowers to the market.

## 1. Introduction

*Panax ginseng* C.A. Meyer belongs to the genus *Panax* in the family Araliaceae, and its roots have been used as a natural medicine for thousands of years in Asian countries, most notably in China, Korea, and Japan [1]. It has become one of the most popular and bestselling herbs in the global herb market [2,3]. Korea is currently the second-largest producer and exporter of ginseng roots after China [4]. Ginseng saponins (ginsenosides) are known to be the main bioactive agents with various pharmacological features and health-promoting attributes [5,6,7], including anti-aging [8], anti-stress [9], anti-oxidative [10], anti-fatigue [11], anti-diabetes [12], anti-cancer [13], enhanced liver function [14], improved immune system [15], improved climacteric disorder response, and sexual function [16]. Nearly two hundred ginsenosides have thus far been isolated and identified from a variety of tissues of ginseng plants [17]. Based on the various chemical structures of aglycone moieties, ginsenosides are mostly divided into protopanaxadiol (PPD) types, such as Rb1, Rb2, Rc, Rd, or Rg3; and proa topanaxatriol (PPT) types, like Rg1, Re, Rg2, or Rh1. The effectiveness of ginseng for a variety of health situations makes it a popular choice as a health product, dietary supplement, food, and cosmetic product [18]. Moreover, ‘ginseng’ in the literature and media refers to the root of ginseng, unless otherwise stated.

At present, phytochemical studies have revealed that ginseng leaves contain abundant ginsenosides, and the total content of ginsenosides in the leaf is higher than that in the root [19,20,21]. In contrast to those in the ginseng root, ginsenosides Re and Rd are the main ginsenosides in the ginseng leaf [20,21]. In addition, ginseng leaf extract has numerous pharmacological activities that are like those of ginseng root extract [22]. Ginseng leaves have advantages over the roots in terms of cost, source availability, and durability, as ginseng leaves can be harvested every year, while the root typically takes four to six years to grow. At the very least, ginseng leaves may serve as a valuable source of Re and Rd ginsenosides [23,24].

Traditionally, the leaves of *P. ginseng* are mostly consumed as tea [25]. The majority of ginseng leaf tea produced in Korea is exported, and studies of its hygienic safety and quality have been reported [26,27]. During the cultivation of ginseng, flowers are usually used as a healthy tea due to their medicinal potential such as anti-fatigue and immune enhancement, which serve as evidence for their prospective use in functional foods. In the roots and flowers of ginseng, saponins and polysaccharides are the major active constituents [28,29,30,31,32,33]. In recent years, owing to the popularity of barbecues in South Korea, ginseng leaves have become a new component of barbecues, regarded as a popular high-end way to eat them. Furthermore, salads with ginseng flowers have also become very popular. It is thus evident that there is still room for the development of ginseng leaves and flowers as medicine and food. The cultivation of ginseng is very demanding in terms of the light environment, as it is a shade-loving crop that is traditionally grown under a thatch of impermeable straw in Asia [34]. The response of ginseng plants to the light environment may be characterized as follows: too little light reduces the yield of roots [35]; and too much light results in the photoinhibition of photosynthesis, photobleaching, and the death of leaves [36,37,38,39]. Ginseng leaves sparsely erect trichomes on the adaxial surface. Trichomes are known to respond to abiotic environmental factors such as salinity, drought, elevation, and light to adapt to the growth environment [40,41,42]. An increase in the light intensity resulted in a significant increase in the trichome density, which was affected by the photoperiod and temperature in *Solanum habrochaites* [43].

Additionally, ginseng flower formation is also highly associated with light. Since most studies have been focused on light intensity or duration, scientists are relatively less attracted to the subject of lighting direction. The lighting direction is related to phototropism, which is the orientation of the plant growth direction in response to light. This effect is universal in green elongating plants, and the response is sensitive in seedlings, which could curve toward or away from the light source [44]. Changing the lighting direction not only affects the morphology of plants, including shoot length; stem diameter; leaf size, number, and thickness; and flower bud formation and number, but also the plant physiology [45,46,47]. Apart from light factors, plant lodging also significantly affects the yield and quality of ginseng crops and makes harvesting them difficult [48]. Overall, selecting a suitable light environment, changing the ginseng morphology, reducing lodging loss, and increasing yield are the key points of ginseng cultivation.

The current study aimed to investigate the effect of lighting direction on ginseng morphology and physiology with the aim of obtaining sprouted plants with short and thick shoots with enlarged leaves and strong roots. Moreover, no other external application of hormones, fertilizers, or chemicals was considered in this study; we simply changed the direction of light, which is innovative and environmentally friendly and provides a new solution for ginseng cultivation.

## 2. Results

### 2.1. Morphology and Growth Parameters

**Figure 1:**
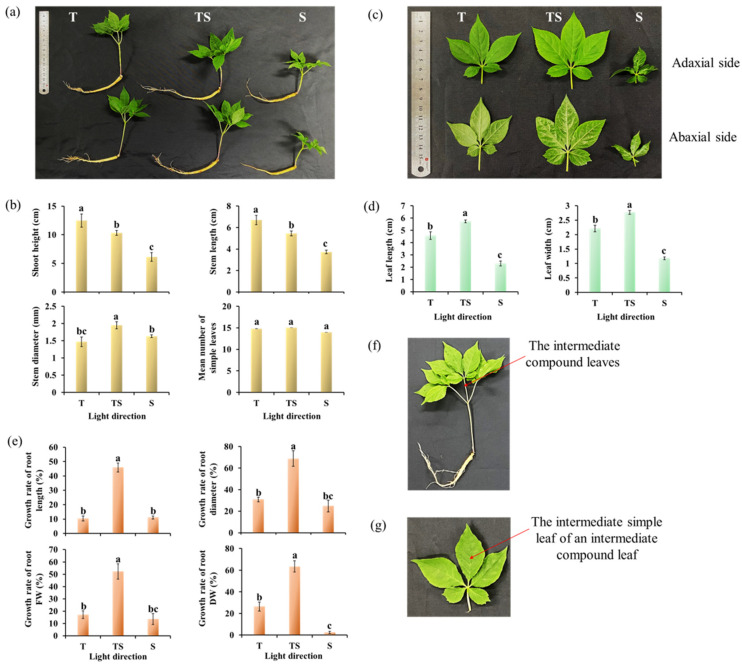
Morphology (**a**,**c**), morphologic parameters (**b**,**d**), and the relative growth rate (**e**) of ginseng shoots, leaves, and roots as affected by the different lighting directions for 21 days. T, top; TS, top + side; S, side. Leaves in (**c**) are the intermediate compound leaves (**f**) of ginseng plants. Leaf length and width were measured according to the intermediate simple leaf of an intermediate compound leaf (**g**). The lowercase letters indicate significant separation within treatments by the Duncan’s multiple range test at *p* ≤ 0.05 in the same cultivar. Vertical bars indicate the means ± standard error (*n* = 12).

The lighting direction significantly affected the morphology in Korean ginseng (Figure 1 and Table 1). After 21 days of cultivation, the plants under the TS lighting displayed shortened shoots, enlarged leaves, and thick stems as compared to the ones grown under T or S lighting (Figure 1a,c). The shortest ginseng shoots were obtained with the side lighting, but with small, irregular, and wrinkled leaves (Figure 1a,c), which was inconsistent with the purpose of this study. Additionally, the TS lighting was more conducive to root growth and development, contributing to the longest and thickest ginseng roots (Figure 1a,e). Although the lighting direction significantly affected the shoot height, stem thickness, leaf size, and root growth, it did not affect the leaf number of ginseng plants (Figure 1b).

The TS lighting significantly enhanced the shoot growth and development, leading to the greatest fresh and dry weights (Table 1). Moreover, compared with the roots before treatment, the TS lighting was the most beneficial among the three treatments for root development, which resulted in the sprouted plants with the best growth traits, including the growth, length, diameter, and FW or DW of ginseng roots (Figure 1e and Table 1). Another point that needed to be considered was carbon allocation in the various lighting direction treatments. Clearly, the allocation of the shoot DW in the S lighting treatment was an order of magnitude lower than the root DW, indicating a relocation to the roots in this treatment. Notably, the allocation in the other treatments was comparatively similar between roots and shoots. Moreover, there was almost no difference between the pre- and post-treatment root DW in the S lighting treatment (Table 1).

### 2.2. Micro-Observation of Trichomes and Leaf Thickness

**Figure 2:**
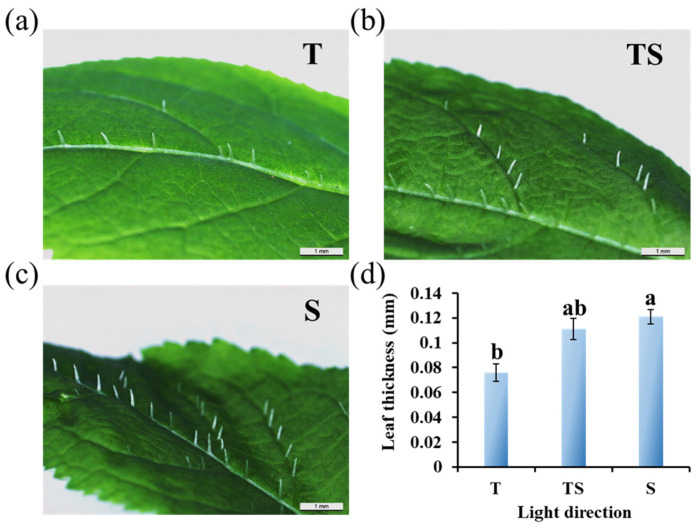
Epidermal hair micro-observation (**a**–**c**) and thickness (**d**) of ginseng leaves as affected by the different lighting directions for 21 days. T, top; TS, top + side; S, side. Leaf epidermal hair micro-observations and thicknesses were based on the intermediate simple leaf of an intermediate compound leaf (as shown in Figure 1g). The lowercase letters indicate significant separation within treatments by the Duncan’s multiple range test at *p* ≤ 0.05 in the same cultivar. Vertical bars indicate the means ± standard error (*n* = 12).

The side lighting resulted in the thickest leaves with the greatest number of thicker trichomes on the adaxial side of ginseng leaves, followed by the TS lighting. At the same time, ginseng plants grown under top lighting had the smoothest and thinnest leaves with weak trichomes (Figure 2).

### 2.3. Flower Bud Formation

**Figure 3:**
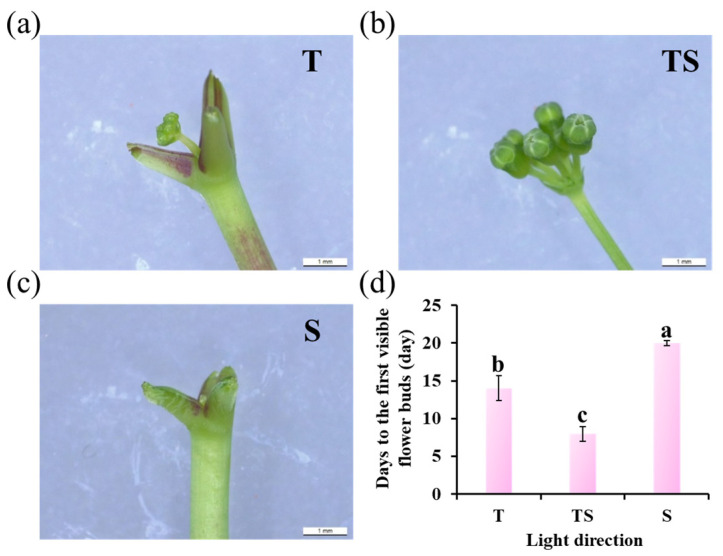
Flower bud state observation (**a**–**c**) and days (**d**) to the first visible flower bud in ginseng plants, as affected by the different lighting directions for 21 days. T, top; TS, top + side; S, side. The lowercase letters indicate significant separation within treatments by the Duncan’s multiple range test at *p* ≤ 0.05 in the same cultivar. Vertical bars indicate the means ± standard error (*n* = 12).

The TS lighting significantly enhanced the appearance of the first flower bud, followed by the top lighting. The side lighting seemed to inhibit the formation of flower buds to a certain extent, leading to the last flower bud observation (Figure 3).

### 2.4. Photosynthesis-Related Pigment Contents

**Figure 4:**
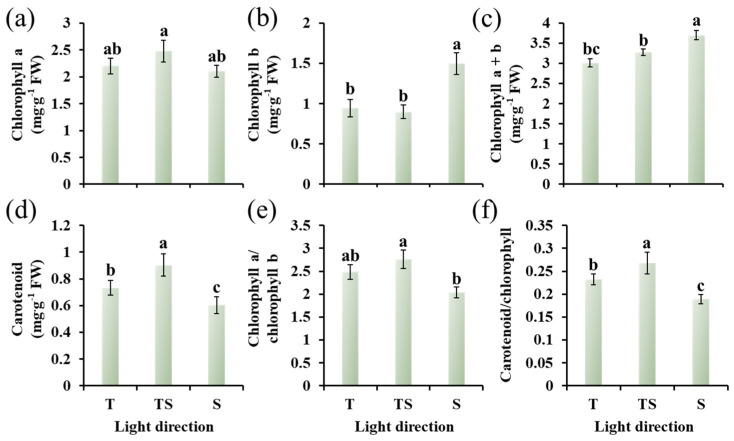
The photosynthesis-related pigments in ginseng leaves as affected by the different lighting directions for 21 days. Chlorophyll a (**a**), chlorophyll b (**b**), chlorophyll a + b (**c**), carotenoid (**d**), chlorophyll a/chlorophyll b (**e**), and carotenoid/chlorophyll (**f**). T, top; TS, top + side; S, side. The lowercase letters indicate significant separation within treatments by the Duncan’s multiple range test at *p* ≤ 0.05 in the same cultivar. Vertical bars indicate the means ± standard error (*n* = 12).

The TS lighting direction was the most favorable to the biosynthesis of some photosynthesis-related pigments, such as chlorophyll a and carotenoid, followed by the top lighting (Figure 4a,d). Meanwhile, the ratios of chlorophyll a to chlorophyll b and carotenoid to total chlorophyll increased in the TS lighting (Figure 4e,f). However, the total chlorophyll content was slightly decreased in response to the TS lighting than to the side lighting (Figure 4c), which might have been caused by the sharply decreased content of chlorophyll b (Figure 4b).

### 2.5. Photosynthetic and Chlorophyll Fluorescence Characteristics

As shown in Table 2, the TS lighting resulted in the best values of *P*n, *G*s, and *C*i, followed by the top lighting, while the worst values were observed in response to the side lighting. The *T*r was lower in response to the TS lighting and significantly lower in response to the side lighting.

The *F*v/*F*m was not affected by the light coming from the T or TS but was reduced by the side lighting (Table 2). Under non-stress conditions, the change in this parameter was very small, which was not affected by the species nor the growth conditions, while under stress conditions, the *F*v/*F*m decreased significantly. Still, the TS lighting caused the best value of *F*v′/*F*m′ and *qP*, followed by the top lighting. However, the worst value of NPQ appeared in response to the TS lighting, followed by the top lighting. The greatest NPQ observed in response to the side lighting may have been caused by the increased heat dissipation capacity.

## 3. Discussion

### 3.1. Shortened Shoots, Enlarged Leaves, and Strong Roots

When compared with the top or side lighting, the TS lighting shaped ginseng plants with short, thick shoots; enlarged leaves with more trichomes; and strong roots (Figure 1 and Figure 2 and Table 1), which is consistent with the previous research, which found that TS lighting promotes the growth and development of chrysanthemums [47]. Leaf orientation is a direct determinant of light interception. Variations in the leaf angle and leaf movement due to phototropism (epinasty or hyponasty) have been proposed to increase the photosynthetic capacity, efficiency, and carbon gain under competitive conditions for light [49,50,51]. The TS lighting remarkably increased the fresh and dry shoot weights but decreased the shoot length, which agrees with the results of earlier research in which the sideward lighting induced considerably shorter stems but increased the dry weight of in vitro micropropagated potato plantlets when compared to those grown with top lighting [52]. One more point which needed to be considered was carbon allocation as affected by lighting direction. As shown in Table 1, a significant difference was found between the DW of shoots and roots in the S lighting treatment, indicating a relocation to the roots in this treatment. With regard to plant carbon allocation, the source–sink hypothesis holds that plant carbon allocation is based on a series of laws linking carbon sources (mainly leaves) and pools (mainly stems, roots, and fruits) [53]. Carbon allocation depends on the supply capacity of the source, the competitiveness of the reservoir, and the transport capacity of the stem to the photosynthetic products. The functional balance hypothesis holds that the growth of above-ground parts of plants is limited by the rate of carbon fixation via photosynthesis, and the growth of roots is limited by the rate of water and nutrient uptake by roots [54]. Light environment usually changes the demand for other resources by affecting the photosynthetic intensity of plant leaves [55]. The interaction between light and soil water and nutrients also significantly affects the distribution of plant photosynthetic products [56,57]. Therefore, when the light environment is not suitable or the water and nutrients are insufficient, the plant’s photosynthetic products are more distributed to the root system [54]. It is also possible that the larger stem diameter and well-developed roots of ginseng observed in response to the TS lighting are upregulated by higher photosynthetic rates, which provides adequate energy for the shoots and roots [58] by combining endogenous plant hormones with the complex molecular regulatory networks [59,60].

Moreover, the TS and S lighting most significantly increased the leaf thicknesses (Figure 2). High light interception ability and photosynthetic efficiency are provided by large leaf areas. The photosynthetic rate is affected by the leaf area and the amount of carbon partitioned into thicker leaves, which further contributes to the development of foliar structures [61,62]. Plants have trichomes on the surfaces of their leaves, which respond to abiotic environmental factors such as salinity, drought, elevation, and light, to adapt to the growth environment [40,41,42]. The presence of trichomes strengthens the protective role of the epidermis. On the one hand, it provides relative protection against biological aggression. On the other hand, it weakens the influence of strong light and strengthens the control of transpiration, which is beneficial for plant life. Increasing the light intensity has been shown to significantly increase the trichome density in some cases [43]. Plant trichomes are also of high application and economic value. In some plants, secretory glandular hairs are present, and these hairs can synthesize, store, and secrete a variety of metabolites, including organic acids, polysaccharides, proteins, polyphenols, alkaloids, and terpenoids [63,64,65,66,67]. They are responsible for giving a unique smell to plants; can be refined into fragrances, medicines, pesticides, food additives, resins, and essential oils; and are of great commercial value. For this reason, plant trichomes are known as mini-chemical plants for the generation of high-value natural products [65,68]. Examples include artemisinin, an antimalarial drug extracted from *Artemisia annua*; menthol, which is synthesized from the trichomes of *Mentha* spp.; and cannabinoids, which are the active ingredient in *Cannabis sativa* [63,68]. Li et al. (2005) showed that the trichomes of the fern *Pteris vittata* can take up and store arsenic from soils, which also provides a novel insight for the management of heavy metal pollution in soils [69]. Plant lodging resistance is highly associated with plant height, fresh weight, stem diameter, and other parameters [70,71]. Based on this study, the combined light direction (TS lighting) shaped ginseng plants with enhanced morphological characteristics of shorter height, thicker stem, and the greatest fresh weights of the shoots and roots, which are important characteristics in improving the plants resistance against the lodging. This preliminary research may provide new ideas and orientations regarding lodging resistance and increase production in ginseng sprouts.

### 3.2. Early Flower Bud Formation

The current research showed that the TS lighting significantly promoted earlier flower bud formation in ginseng plants when compared to the top lighting and especially the side lighting, which appeared to inhibit flower bud formation to some degree and led to the late observation of flower buds (Figure 3). This promotion, by the TS lighting, of flower bud formation is in accordance with our previous study that showed TS lighting leads to excellent performance in the flowering of chrysanthemums [47]. The photoperiodic pathway, vernalization pathway, temperature pathway, autonomous pathway, gibberellin pathway, and age pathway have all been found in plants as flowering regulating processes. In the case of leaves, light signals are detected by phytochromes, cryptochromes, or ZTL/FKF1/LKP2, which are then transmitted to the circadian clock. Finally, photoreceptors regulate flowering either directly or indirectly after signals integrate through a variety of flowering pathways [72,73,74,75]. Multiple photoreceptors capable of responding to various wavelengths of light are located on the upper surface of the leaf. The resulting regulators are then transferred from the phloem to the apical meristem, where they combine with a suite of proteins to produce a transcriptionally active flowering complex that triggers flowering [76].

Ginseng plants cultured with the TS lighting induced the greatest number of leaves with a larger leaf size that can effectively capture and utilize the available light, as well as promote the expression of flowering-related genes. In addition, the TS lighting resulted in the highest luminous efficiency. In this experiment, plants grown under the TS lighting exhibited the greatest flower bud formation, indicating that these buds received sufficient light, metabolized vigorously, grew cells rapidly, and thus preferentially received more nutrients. Overall, adjusting the contact area between the top surface of the leaf and the lumen is an important factor for efficient light utilization [77]. The TS lighting induced flower bud formation ahead of time by providing more favorable conditions. This may explain why TS lighting exerted such a large positive influence on flowering.

### 3.3. Photosynthetic and Chlorophyll Fluorescence Characteristics

In this study, the TS lighting substantially enhanced the *P*n, *T*r, *G*s, and *C*i levels in ginseng (Table 2). Improvements in the photosynthetic traits led to the increased carbon gain and growth of chrysanthemums [78]. In addition, well-developed leaf structures and an abundance of chlorophyll were closely associated with the increased net photosynthetic rate in ginseng in response to the TS lighting [79,80,81]. Multiple thick trichomes strengthen the protective effects of the epidermis and weaken the effects of strong light and enhance the control of transpiration, thus protecting the basic physiological activities of the plant.

The greater the number of electrons flowing through PSII, the greater the photosynthetic capacity [82]. The fluorescence properties of chlorophyll are the most important component in the regulation of photosynthesis and plant responses to environmental variables because of its sensitivity and observability [83]. Many photosynthetic processes are intimately linked to chlorophyll fluorescence characteristics, and the effects of any stress on a specific process of photosynthesis can be represented by the kinetics of chlorophyll fluorescence [84]. A positive linear association between the fluorescence traits and chlorophyll concentration in the leaves of living plants has been found in previous research [85]. A similar result was found in this research, in which improvements in the chlorophyll fluorescence characteristics were recorded in the ginseng plants in response to the TS lighting (Table 2). This result shows that an optimal combination of lighting directions upgrades the PSII proficiency and, as a result, could further improve photosynthesis by advancing the energy transport from PSII to PSI.

### 3.4. Further Research

According to this preliminary study of how the lighting direction affects ginseng morphology, the TS lighting was optimal in regulating ginseng growth and development, which resulted in short, thick shoots; enlarged, thick leaves; strong, thick roots; and earlier flower bud formation. However, the mechanisms underlying these phenotypic changes still need to be further explored: certain gene regulations at the molecular level and the biosynthesis of plant hormones that are related to plant growth, specific leaf area, leaf structures, and flowering. More important is the variation in the biosynthesis and content of healthy compounds in ginseng roots, stems, leaves, and flowers. This current study provides a new research idea for phenotypic improvements as directed by the lighting conditions for ginseng cultivation.

## 4. Conclusions

In a conclusion, the lighting direction significantly influenced the morphophysiology of Korean ginseng. Compared with the common single top artificial lighting, the combination of top and side lighting appeared as the optimal lighting direction, which was more effective in improving ginseng growth and development, as indicated the relative growth rate of shoots and roots, specific leaf area, flower bud formation, biosynthesis of photosynthetic pigments, and photosynthesis characteristics. And the single side lighting resulted in the worst growth parameters and seemed to inhibit the formation of the flower buds to a certain extent, leading to the latest flower bud observation. In addition, the current study found that the side lighting was a positive factor in increasing the leaf thickness and number of trichomes on the leaf adaxial surface. However, the participation of the top lighting weakened these traits. Taken together, the composite light direction (TS lighting) shaped ginseng plants with enhanced characteristics of short, thick shoots; enlarged, thick leaves; more leaf trichomes; earlier flower bud formation; and improved photosynthesis. Combined with the current cultivation of and market demand for ginseng, this preliminary research may provide new ideas and orientations in ginseng cultivation lodging resistance and in improving the supply of ginseng roots, leaves, and flowers to the market. No other external application of hormones, fertilizers, or chemicals took place in this research, which is more in line with a green sustainable development strategy. In further studies, the plant-hormone- or molecular-mediated regulatory systems involved in these phenotypic changes need to be explored in depth.

## 5. Materials and Methods

### 5.1. Plant Materials and Growth Conditions

**Figure 5:**
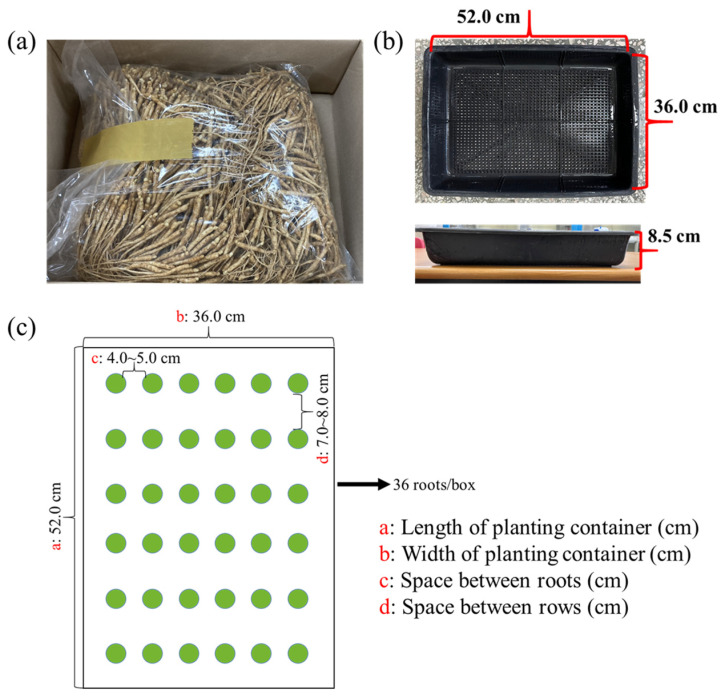
One-year-old roots of Korean ginseng (**a**); top and side views of the rectangular planting container (length 52.0 cm × width 36.0 cm × height 8.5 cm) (**b**); planting pattern (**c**).

One-year-old ginseng roots featuring similar morphologies to a main taproot and a tiny emerging shoot were obtained from a ginseng farm in Geumsan, Chungnam, Republic of Korea, in early August 2022 (Figure 5a) and kept at 4 °C until use. Again, before formally beginning the experiment, the roots were carefully chosen to ensure consistency in the root shape, size, and especially weight. In rectangular planting containers, the selected roots were pinned in a commercial medium (BVB Medium, Bas Van Buuren Substrates, EN-12580, De Lier, The Netherlands) (Figure 5b). At planting time, the thickness of the medium was kept fundamentally equal to the height of the container. Following planting, the detailed planting scheme of 36 roots per container was used, as shown in Figure 5c. And then, the roots were transferred to plant growth chambers (C1200H3, FC Poibe Co., Ltd., Seoul, Republic of Korea) for 3 to 5 days of dark adaptation with a temperature of 20 °C and a relative humidity of 45% to 50%. The first watering was ensured to be thorough. The plants were irrigated daily with a multipurpose nutrient solution (macro-elements: Ca^2+^, Mg^2+^, K^+^, NH_4_^+^, NO_3_^−^, SO_4_^2−^, and H_2_PO_4_^−^; microelements: B, Cu, Fe, Mn, Mo, and Zn; pH = 5.5–6.0) [45]. Additionally, this study was not only designed with a completely randomized layout but also had 108 biological replications per treatment with consistent growth to minimize external influences.

### 5.2. Lighting Treatments

To establish a light environment for seedling production, we investigated the effects of the light intensity and photoperiod, as well as their combination as daily integrals of light, on the growth and physiological traits of *Panax ginseng* seedlings. According to Lee at el. (2022), a light intensity of 50 μmol m^−2^ s^−1^ PPFD with a 12 h d^−1^ photoperiod was a suitable light environment for both the shoot and root growth of ginseng seedlings [86]. However, the ginseng sprout grower (Dream Farm, Sacheon, Republic of Korea) used 30 μmol m^−2^ s^−1^ PPFD with a 12 h d^−1^ photoperiod. In order to maintain the consistency of the ginseng growing environment as much as possible, the light intensity and photoperiod conditions in this study were the same as those of the ginseng sprout grower (Dream Farm, Sacheon, Republic of Korea).

**Figure 6:**
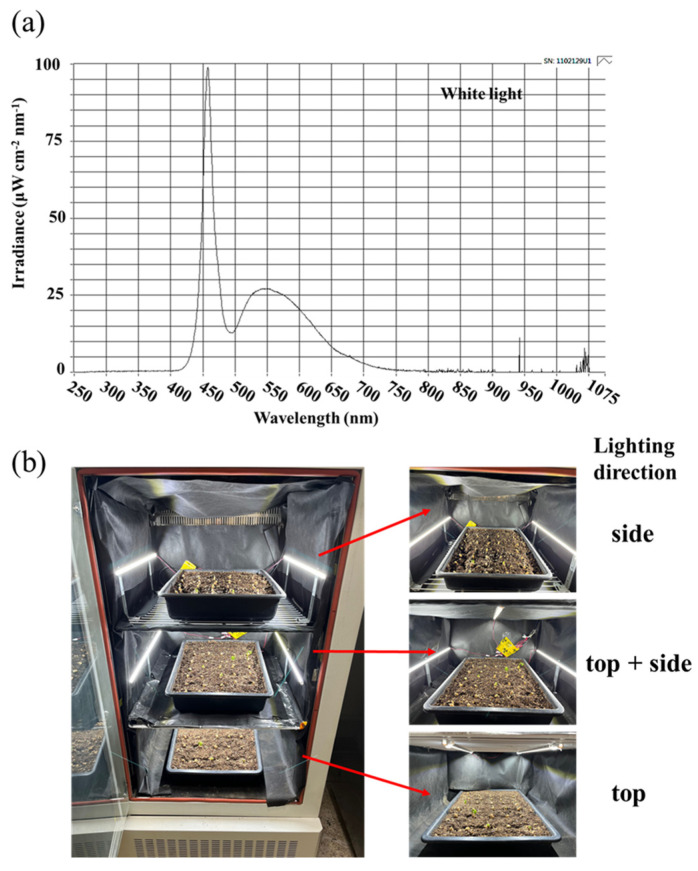
The spectral distribution of the experimental light treatments (**a**): the daily white light (~400–720 nm, peaked at 435 nm) provided by white LEDs; the experimental layout and lighting direction design employed in this study (**b**). Side, S; top + side, TS; and top, T.

After the dark acclimation, still in these growth chambers, with all other parameters being equal, the light processing was started with a 12 h d^−1^ photoperiod every day from 8:00 a.m. Plants were grown with an incident light intensity of 30 ± 5 μmol·m^−2^·s^−1^ PPFD provided by white MEF50120 LEDs (More Electronics Co. Ltd., Changwon, Republic of Korea) with a wide spectrum ranging from 400 to 720 nm and a distinct peak at 435 nm in blue (Figure 6a). And these two modular-type LED lamps were placed 25 cm away from the top or 20 cm away from the side of the plants to form three lighting direction treatments, which were the top, side, and top + side (Figure 6b). The pulse width control method (PWM) LED dimmer was used separately in different directions to maintain the consistency of light intensity in each treatment and ensure that ginseng plants were exposed to a light intensity of 30 ± 5 μmol·m^−2^·s^−1^ PPFD.

A total of three chambers and three repetitions were used. Each chamber was divided equally into three compartments using plates according to the lighting direction. The lighting direction was randomized within each chamber to avoid positional effects. All portions reflecting light within the chambers, as well as the plates in each layer, were enclosed in an opaque black curtain to prevent light from interacting with one another. The distribution of light was recorded at 1 nm wavelength intervals using a spectroradiometer (USB 2000 Fiber Optic Spectrometer, Ocean Optics Inc., Dunedin, FL, USA; detection wavelength between 200 nm and 1000 nm), and the uniformity was checked by measuring the intensity of the light at three points in each canopy-level light treatment with a quantum radiation probe (FLA 623 PS, ALMEMO, Holzkirchen, Germany).

### 5.3. Measurement of the Growth Parameters, Calculation of the Relative Growth Rate, and Observation of the Leaf Trichomes and Flower Buds

Repeated experimentation allowed us to extend the experimental duration to 21 days to ensure that three compound ginsengs leaves were fully expanded for each lighting direction. After 21 days of the light treatments, plant growth parameters such as the plant height, shoot diameter, shoot length, leaf number, and flower buds per plant were collected. The days to visible flower buds in each treatment were determined by counting the number of days from initiation of the light treatments to the date when the first flower bud appeared. The diameter of the stem was measured based on the middle portions of the main stem. The length and width of the leaves were based on the single intermediate leaf of an intermediate compound leaf. To measure the biomass, after thorough cleaning, split shoot and root samples were oven dried (drying oven, Venticell-222, MMM Medcenter Einrichtungen GmbH., Munich, Germany) at 85 °C for 5~7 days until a constant mass was achieved to determine the dry mass. Harvested samples were also kept in liquid nitrogen immediately and then stored in a refrigerator at −80 °C for the subsequent physiological studies.

The relative root growth rate consisting of the fresh weight and diameter was calculated after the plants were harvested. The fresh weight and ginseng root diameter were recorded individually prior to planting. Once all the data on fresh weight, dry weight, length, and diameter were obtained, the relative growth rate of the roots in these parameters was calculated using the following formula: Relative growth rate (%) = (harvested value-original value)/original value × 100% (*n* = 12) of roots.

The microscopic observation and thickness determination of the leaf epidermal hairs were performed on the single intermediate leaf of an intermediate compound leaf (as shown in Figure 1g). After 21 days of cultivation, the leaf adaxial side was directly observed with an optical microscope (ECLIPSE Ci-L, Nikon Corporation, Tokyo, Japan) (magnification 20×), and the leaf thickness was analyzed with ImageJ (ImageJ 1.48v, NIH, Bethesda, MA, USA). The magnification for viewing the ginseng flower buds was 5×.

### 5.4. Measurement of the Photosynthetic Pigment Contents

The chlorophyll and carotenoid contents of the leaves were determined and calculated as reported by Lichtenthaler and Buschmann (2001) [87]. At the end of the 21 days of the lighting treatments at 9:00 a.m., 0.2 g of fresh leaf sample was taken from the intermediate single leaf of an intermediate compound leaf (as shown in Figure 1g) and grinded using liquid nitrogen and extracted in 2 mL of 80% acetone (*v*/*v*) overnight at 4 °C until the leaf samples were completely decolorized. Colorimetry was performed at A_470nm_, A_646nm_, and A_663nm_ using a UV spectrophotometer (Libra S22, Biochrom Ltd., Cambridge, UK).

### 5.5. Measurement of Photosynthesis and Chlorophyll Fluorescence

The *P*n, *T*r, *G*s, and *C*i of the intermediate simple leaf of an intermediate compound leaf (as shown in Figure 1g) in each plant was measured with a leaf porometer (SC-1, Decagon Device Inc., Pullman, WA, USA) at the harvest time. Measurements were made at four positions on each sheet, and the average result was used. From 9:00 to 11:00 a.m., these parameters were measured in a closed-type plant factory to keep the same steady condition and avoid measurement errors caused by changes in the light environment.

A photosystem (Fluor Pen FP 100, Photon Systems Instruments, PSI, Drásov, Czech Republic) was used to measure the chlorophyll fluorescence in the leaves. As above, the single intermediate leaf from an intermediate compound leaf from each plant was chosen for these measurements. Leaves were dark-adapted using a leaf clip for 30 min and then given a saturating light pulse of 0.6 s (3450 μmol·m^−2^·s^−1^ PPFD) to obtain the maximum (*F*m) and minimum (*F*0) fluorescence. The leaves were then light-adapted with 5 min of continuous actinic light (300 μmol·m^−2^·s^−1^ PPFD, as in the growth condition) with saturating pulses every 25 s, after which, the maximum light-adapted fluorescence (*F*m′) and the steady-state fluorescence (*F*s) were recorded. The *F*v/*F*m was calculated to be *F*v/*F*m = (*F*m − *F*0)/*F*m [88]. After excitation with PSI (*F*0′), the actinic light was turned off and a far-red pulse was applied to achieve minimal fluorescence. And the *F*v′/*F*m′ = (*F*m′ − *F*s)/*F*m′ was used to calculate the *F*v′/*F*m′. In addition, the *qP* was calculated to be *qP* = (*F*m′ − *F*s)/(*F*m′ − *F*0′) [89].

### 5.6. Statistical Analysis

All plants used in the current study were sampled at random. Data processing, plotting, and statistical analysis were performed in Excel 2016 and the DPS package (DPS for Windows, 2009). Analysis of variance (ANOVA) was used to assess significant differences between the treatments, followed by Duncan’s multiple range test at a probability (*p*) ≤ 0.05 with the aid of a statistical program (SAS, Statistical Analysis System, V. 9.1, Cary, NC, USA). Differences between each treatment were tested using Student’s t test (*p*) ≤ 0.05. In addition, 12 biological replicates were carried out to obtain all results, including each measurement, calculation, or observation, which are presented as mean ± standard error.

## Figures and Tables

**Table 1:** Growth parameters of ginseng plants before or after 21 days of the lighting direction treatments.

**Light** **Direction ^1^**	**Shoot FW ^2^** **(g)**	**Shoot DW ^3^** **(g)**	**Pre-Root FW** **(g)**	**Post-Root FW** **(g)**	**Pre-Root DW** **(g)**
T	0.793 ± 0.011 b ^4^	0.124 ± 0.008 b	0.714 ± 0.094	0.836 ± 0.021 b	0.121 ± 0.006
TS	1.075 ± 0.017 a	0.187 ± 0.010 a		1.087 ± 0.014 a	
S	0.372 ± 0.020 c	0.056 ± 0.007 c		0.797 ± 0.032 bc	
**Light** **Direction**	**Post-Root DW** **(g)**	**Pre-Root Length** **(cm)**	**Post-Root Length** **(cm)**	**Pre-Root Diameter** **(mm)**	**Post-Root Diameter** **(mm)**
T	0.153 ± 0.012 b	10.871 ± 1.023	12.004 ± 1.147 b	3.164 ± 1.001	4.142 ± 0.987 b
TS	0.198 ± 0.010 a		15.863 ± 1.263 a		5.341 ± 1.000 a
S	0.124 ± 0.009 c		12.075 ± 1.536 b		3.790 ± 1.023 bc

^1^ T, top; TS, top + side; S, side. ^2^ Fresh weight. ^3^ Dry weight. ^4^ Mean separation within columns by the Duncan’s multiple range test at *p* ≤ 0.05, and the values are average ± standard error (*n* = 12). Pre-root FW, DW, length, and diameter were measured before the treatments; post-root FW, DW, length, and diameter were measured after the treatments.

**Table 2:** Photosynthetic indexes and chlorophyll fluorescence parameters of ginseng plants as affected by the lighting direction after 21 days.

**Light Direction ^1^**	***P*** **n ^2^** **(μmol CO_2_ m^−2^** **·** **s^−1^)**	***T*** **r ^3^** **(mmol H_2_O m^−2^** **·** **s^−1^)**	***G*** **s ^4^** **(mol H_2_O m^−2^** **·** **s^−1^)**	***C*** **i ^5^** **(μmol CO_2_ mol^−1^)**
T	12.137 ± 0.351 b ^10^	1.893 ± 0.084 a	0.437 ± 0.021 b	438.337 ± 15.119 b
TS	15.004 ± 0.435 a	1.542 ± 0.071 b	0.586 ± 0.037 a	486.274 ± 13.996 a
S	10.016 ± 0.375 c	1.165 ± 0.080 c	0.368 ± 0.023 c	404.158 ± 14.772 c
**Light Direction**	***F*** **v/*F*m ^6^**	***F*** **v′/*F*m′ ^7^**	***qP*** **^8^**	**NPQ ^9^**
T	0.860 ± 0.011 a	0.526 ± 0.010 b	0.425 ± 0.009 b	2.625 ± 0.067 ab
TS	0.862 ± 0.012 a	0.597 ± 0.018 a	0.473 ± 0.012 a	2.347 ± 0.074 b
S	0.812 ± 0.007 b	0.523 ± 0.011 b	0.353 ± 0.023 c	2.801 ± 0.083 a

^1^ T, top; TS, top + side; S, side. ^2^ *P*n, net photosynthetic rate. ^3^ *T*r, transpiration rate. ^4^ *G*s, stomatal conductance. ^5^ *C*i, intercellular CO_2_ concentration. ^6^ *F*v/*F*m, the maximum PSII quantum yield. ^7^ *F*v′/*F*m′, the photochemical efficiency of PSII. ^8^ *qP*, the photochemical quenching coefficient. ^9^ NPQ, nonphotochemical chlorophyll fluorescence quenching. ^10^ Mean separation within columns by the Duncan’s multiple range test at *p* ≤ 0.05, and the values are average ± standard error (*n* = 12).

## Data Availability

Data sharing is not applicable to this article.
